# Compound Endoscopic Morphological Features for Identifying Non-Pedunculated Lesions ≥20 mm with Intramucosal Neoplasia

**DOI:** 10.3390/cancers13215302

**Published:** 2021-10-22

**Authors:** João Pedro da Costa-Seixas, María López-Cerón, Anna Arnau, Òria Rosiñol, Miriam Cuatrecasas, Alberto Herreros-de-Tejada, Ángel Ferrández, Miquel Serra-Burriel, Óscar Nogales, Luisa de Castro, Jorge López-Vicente, Pablo Vega, Marco A. Álvarez-González, Jesús M. González-Santiago, Marta Hernández-Conde, Pilar Diez-Redondo, Liseth Rivero-Sánchez, Antonio Z. Gimeno-García, Aurora Burgos, Francisco Javier García-Alonso, Marco Bustamante-Balén, Eva Martínez-Bauer, Beatriz Peñas, Daniel Rodríguez-Alcalde, Maria Pellisé, Ignasi Puig

**Affiliations:** 1 Gastrointestinal Oncology and Endoscopy Research Group, Digestive Diseases Department, Althaia Xarxa Assistencial Universitària de Manresa, 08243 Manresa, Spain; jdacosta@cst.cat; 2 Facultat de Ciències de la Salut, Universitat de Vic-Universitat Central de Catalunya (UVic-UCC), 08243 Manresa, Spain; 3 Gastroenterology Department, Institut de Malalties Digestives i Metabòliques, Hospital Clínic, 08036 Barcelona, Spain; maria.lopez-ceron@salud.madrid.org (M.L.-C.); LRIVERO@clinic.cat (L.R.-S.); 4 Institut d’Investigacions Biomèdiques August Pi i Sunyer, University of Barcelona, 08036 Barcelona, Spain; 5 Clinical Research Unit, Althaia Xarxa Assistencial Universitària de Manresa, 08243 Manresa, Spain; aarnau@althaia.cat; 6 Pathology Department, Althaia Xarxa Assistencial Universitària de Manresa, 08243 Manresa, Spain; orosinol@althaia.cat; 7 Pathology Department, Hospital Clínic, 08036 Barcelona, Spain; mcuatrec@clinic.cat; 8 Banc de Tumors, Biobanc Clinic, Institut d’Investigacions Biomèdiques August Pi i Sunyer, 08036 Barcelona, Spain; 9 Gastroenterology Department, Research Institute Segovia Arana, Hospital Universitario Puerta de Hierro, 28222 Madrid, Spain; alberto.herreros@salud.madrid.org (A.H.-d.-T.); mhconde@salud.madrid.org (M.H.-C.); 10 Digestive Diseases Department, Hospital Clínico Universitario Lozano Blesa, 50009 Zaragoza, Spain; aferrandeza@salud.aragon.es; 11 Centro de Investigación Biomédica en Red de Enfermedades Hepáticas y Digestivas, Biomedical Research Networking Center in Hepatic and Digestive Diseases, 28029 Madrid, Spain; 12 Epidemiology, Biostatistics and Prevention Institute, University of Zurich, 8001 Zurich, Switzerland; miquel.serraburriel@uzh.ch; 13 Digestive Diseases Department, Hospital General Universitario Gregorio Marañón, 28007 Madrid, Spain; oscar.nogales@salud.madrid.org; 14 Digestive Diseases Department, Complexo Hospitalario Universitario de Vigo, 36213 Vigo, Spain; Maria.Luisa.de.Castro.Parga@sergas.es; 15 Digestive Diseases Department, Hospital Universitario de Móstoles, 28935 Madrid, Spain; jlvicente@salud.madrid.org (J.L.-V.); drodrigueza@salud.madrid.org (D.R.-A.); 16 Digestive Diseases Department, Complexo Hospitalario Universitario de Ourense, 32005 Ourense, Spain; PABLO.VEGA.VILLAAMIL@sergas.es; 17 Digestive Diseases Department, Hospital del Mar, 08003 Barcelona, Spain; maalvarez@althaia.cat; 18 Digestive Diseases Department, Complejo Asistencial Universitario de Salamanca, Instituto de Investigación Biomédica de Salamanca (IBSAL), 37007 Salamanca, Spain; jmgsantiago@saludcastillayleon.es; 19 Digestive Diseases Department, Hospital Universitario Río Hortega, 47012 Valladolid, Spain; mdiezre@saludcastillayleon.es; 20 Digestive Diseases Department, Hospital Universitario de Canarias, 38320 Tenerife, Spain; agimenog@ull.edu.es; 21 Digestive Diseases Department, Hospital Universitario La Paz, 28046 Madrid, Spain; burgos.aurora@gmail.com; 22 Digestive Diseases Department, Hospital Universitario de Fuenlabrada, 28942 Madrid, Spain; fgarciaalo@saludcastillayleon.es; 23 Digestive Diseases Department, Hospital Universitario y Politécnico de La Fe, 46026 Valencia, Spain; bustamante_mar@gva.es; 24 Digestive Diseases Department, Corporació Sanitària Parc Taulí, 08208 Barcelona, Spain; EMartinezB@tauli.cat; 25 Digestive Diseases Department, Hospital Universitario Ramón y Cajal, 28034 Madrid, Spain; beatriz.penas@salud.madrid.org

**Keywords:** early colorectal cancer, NBI, optical diagnosis, Paris classification, NICE classification, ESD

## Abstract

**Simple Summary:**

Piecemeal endoscopic mucosal resection (EMR) has proved to be an excellent resection technique for large colorectal polyps. However, a key limitation is the inaccurate histologic assessment of the sample in cases where there is invasion of the submucosa. Thus piecemeal EMR should be avoided if submucosal invasion is suspected. Furthermore, both western and eastern scientific societies have recently recommended that treatment should be based on optical diagnosis (ideally with magnification) which estimates the histology endoscopically. However, experience with magnification in western countries is limited. This study primarily aims to develop a classification system based on endoscopic features to identify intramucosal neoplasia (absence of submucosal invasion) in non-pedunculated lesions ≥20 mm assessed by western endoscopists with narrow band imaging (NBI) and without magnification. We observed that non-ulcerated LST-granular type and LST-non-granular flat elevated lesions represent 58.8% of all non-pedunculated lesions ≥20 mm and are associated with a low risk of submucosal invasion (3.8%). Therefore, we suggest these lesions be treated by piecemeal EMR. In the remaining lesions further diagnostic techniques such as magnifying endoscopy or en bloc resection should be considered.

**Abstract:**

Background: The major limitation of piecemeal endoscopic mucosal resection (EMR) is the inaccurate histological assessment of the resected specimen, especially in cases of submucosal invasion. Objective: To classify non-pedunculated lesions ≥20 mm based on endoscopic morphological features, in order to identify those that present intramucosal neoplasia (includes low-grade neoplasia and high-grade neoplasia) and are suitable for piecemeal EMR. Design: A post-hoc analysis from an observational prospective multicentre study conducted by 58 endoscopists at 17 academic and community hospitals was performed. Unbiased conditional inference trees (CTREE) were fitted to analyse the association between intramucosal neoplasia and the lesions’ endoscopic characteristics. Result: 542 lesions from 517 patients were included in the analysis. Intramucosal neoplasia was present in 484 of 542 (89.3%) lesions. A conditional inference tree including all lesions’ characteristics assessed with white light imaging and narrow-band imaging (NBI) found that ulceration, pseudodepressed type and sessile morphology changed the accuracy for predicting intramucosal neoplasia. In ulcerated lesions, the probability of intramucosal neoplasia was 25% (95%CI: 8.3–52.6%; *p* < 0.001). In non-ulcerated lesions, its probability in lateral spreading lesions (LST) non-granular (NG) pseudodepressed-type lesions rose to 64.0% (95%CI: 42.6–81.3%; *p* < 0.001). Sessile morphology also raised the probability of intramucosal neoplasia to 86.3% (95%CI: 80.2–90.7%; *p* < 0.001). In the remaining 319 (58.9%) non-ulcerated lesions that were of the LST-granular (G) homogeneous type, LST-G nodular-mixed type, and LST-NG flat elevated morphology, the probability of intramucosal neoplasia was 96.2% (95%CI: 93.5–97.8%; *p* < 0.001). Conclusion: Non-ulcerated LST-G type and LST-NG flat elevated lesions are the most common non-pedunculated lesions ≥20 mm and are associated with a high probability of intramucosal neoplasia. This means that they are good candidates for piecemeal EMR. In the remaining lesions, further diagnostic techniques like magnification or diagnostic +/− therapeutic endoscopic submucosal dissection should be considered.

## 1. Introduction

The detection of early colorectal cancer has increased since the introduction of bowel cancer screening programs (BCSP) based on a colonoscopy after a positive fecal immunochemical test (FIT). Forty-six per cent of cancers diagnosed in a BCSP are stage I, and endoscopically resected T1 lesions account for 20% of all colorectal cancers [1].

Large colorectal polyps can be removed by piecemeal endoscopic mucosal resection (EMR), en bloc endoscopic submucosal dissection (ESD) or surgery. Piecemeal EMR has proved to be an excellent resection technique. However, one of its most important limitations is the inaccurate histologic assessment of the sample in the case of invasion of the submucosa (sm). Multiple, poorly-oriented pieces make it difficult to ensure R0 margins, evaluate the depth of invasion, and thus assess the risk factors for lymph node metastasis.

Although endoscopic resection of high-risk T1 colorectal carcinoma (CRC) before surgical resection has no adverse effect on long-term outcomes [2], the limited accuracy of optical diagnosis for predicting sm invasion leads to suboptimal treatment decisions. In the Dutch BCSP, 25% of locally removed T1 CRCs were resected by piecemeal EMR because sm invasion was not suspected. This led to additional surgery in all patients, as the R0 margin and risk factors for LNM could not be assessed [3]. In that study, adjuvant surgery after local treatment was more frequently indicated in patients with T1 CRCs that were not correctly optically diagnosed (41% vs. 11%, *p* = 0.02) [3]. In these cases, ESD would have allowed a more precise histological diagnosis, and additional surgery might have be avoided if none of the risk factors were present. Therefore, although the polyp is amenable to removal by piecemeal EMR, suspicion of sm invasion is crucial before performing the procedure.

The European Society of Gastrointestinal Endoscopy and the US Multi-Society Task Force have recently recommended that treatment should be based on optical diagnosis, not on the endoscopist’s skill [4,5,6]. Japanese guidelines support en bloc endoscopic resection for lesions that might harbour carcinoma, and piecemeal EMR when carcinoma is ruled out with optical diagnosis with magnification [7,8]. However, experience with magnification in western countries is limited.

Previous studies without magnification have already shown that morphology can help to predict which lesions are at higher risk of containing submucosal invasion [9,10,11]. However, all these studies are based on retrospective data and/or regression analysis and can only properly identify a very small subgroup of lesions that can be accurately classified.

Our previous prospective multicentre study, including 2153 lesions >10 mm, found a very stable decision tree for predicting deep sm invasion [12]. The assessment of three features was enough to (1) rule out deep sm invasion and recommend endoscopic treatment in 87% of the lesions; (2) predict deep sm invasion and recommend surgery in 1% of cases; (3) determine lesions with intermediate probability of deep sm invasion that may require further assessment with magnification (12%). However, this study included pedunculated polyps and lesions between 10 and 20 mm, which are more suited to endoscopic removal en bloc, and aimed to predict deep sm invasion in order to recommend surgery.

This study’s primary aim is to develop a classification system based on endoscopic features to identify intramucosal neoplasia (absence of submucosal invasion) in non-pedunculated lesions ≥20 mm assessed by western endoscopists with NBI and without magnification. These lesions may be candidates for piecemeal EMR. Secondary aims were to develop a classification system to identify shallow and deep sm invasion, to be treated with ESD and surgery respectively.

## 2. Materials and Methods

### 2.1. General Study Design and Site

Post-hoc analysis of an observational prospective multicentre study was conducted at 17 academic and community hospitals by 58 endoscopists. The main results for predicting deep sm invasion in lesions >10 mm have been already published [12]. As in the previous study “the Standards for Reporting of Diagnostic Accuracy recommendations were followed. The protocol was registered in ClinicalTrials.gov (NCT02328066) and was approved by the local ethics committee (Code number CEIC14/47). Patients provided written informed consent before inclusion. Study data were collected and managed using REDCap electronic data capture tools hosted at the Asociación Española de Gastroenterología website (www.aegastro.es, accessed on 1 June 2014) (Supplementary Document S1)” [12].

### 2.2. Participants and Lesions

All patients scheduled for colonoscopy were consecutively included if a non-pedunculated superficial lesion type 0 in the Paris classification (not obvious cancer) measuring ≥20 mm was diagnosed. Other inclusion criteria were: age >18 years, endoscopic assessment with a high-definition colonoscope with NBI, and written informed consent. Patient exclusion criteria were contraindication for surgical or endoscopic resection, urgent colonoscopy indication, inflammatory bowel disease, and suspected colorectal metastatic disease. Lesion exclusion criteria were obvious cancer, previous biopsy or removal attempt, insufficient bowel cleansing, or histology unavailable [12].

### 2.3. Procedure

All the endoscopists performed a 20-min learning module explaining the NICE classification. During the colonoscopy, the lesion was cleaned and accurately assessed first with white light and then with NBI. Lesion characteristics (size, morphology, gross morphological malignant features (non-lifting sign, chicken skin sign, edge retraction, depressed areas, fold convergence, induration, ulceration, polyp over polyp appearance)), the NICE classification diagnosis and the degree of confidence were recorded. Treatment choice was made in accordance with local practices. For the histology assessment, the local pathologist was informed of the morphology, size, location and resection technique, but was blinded to the optical diagnosis. If a carcinoma was diagnosed (Tis or sm invasion), histology slides were referred for an additional blinded and centralised histology evaluation performed according to the revised Vienna classification [13]. In the case of serrated polyps, the World Health Organisation criteria were applied [14]. The submucosal invasion was measured according to the Japanese guidelines: “when it is possible to identify or estimate the location of the *muscularis mucosae*, depth of sm invasion is measured from the lower border of the muscularis mucosae. When it was not possible to identify or estimate the location of the *muscularis mucosae*, depth of sm invasion was measured from the surface layer [12,15].”

### 2.4. Outcomes

The primary outcome was intramucosal neoplasia. According to the Vienna [13] and the WHO [14] classifications, intramucosal neoplasia includes serrated lesions, low-grade neoplasia (LGN) and high-grade neoplasia (HGN). HGN also includes high-grade dysplasia and intramucosal carcinoma (Tis). Secondary outcomes were shallow (<1 mm) and deep sm invasion (≥1 mm)

### 2.5. Statistical Analysis

Categorical variables are presented as absolute values and relative frequencies. Continuous variables are summarised as means and standard deviations (SDs) or as medians and interquartile range in the case of non-normal distributions.

The variables associated with deep invasion were examined using bivariate analysis. Student’s *t*-test or the non-parametric Mann–Whitney test were used for continuous variables. Chi-square test, Fisher’s exact test, or the Monte Carlo method (in 2 × 2 contingency tables or *n* × 2, where the expected frequencies were <5) were used for categorical variables.

Unbiased conditional inference trees (CTREE) were fitted to identify intramucosal neoplasia, including the lesions’ optical characteristics. CTREE is a conditional recursive partitioning algorithm that solves both the overfitting problem and the variable selection bias present in other recursive partitioning algorithms. This methodology aims to maximise the predictive power through relevant interaction detection, while keeping a simple and clinically relevant structure. All the variables are potential candidates for inclusion in the model. Compared to classical multivariable analysis (i.e., logistic regression), the variable selection process is automated, and no assumptions regarding the underlying structure and distribution are needed. As a result, the tree shows the variables in a hierarchical structure of the model that has a relative real weight for taking decisions, not only statistically significant ones. This approach is thus more aligned with human decision making when facing a problem of clinical diagnosis. The conditional inference tree methodology is described in more detail in the Supplementary Document S2 [16,17,18].

The level of statistical significance was 2-sided 5% (*p* < 0.05). For the statistical analysis, STATA, version 14 (StataCorp LP, College Station, TX, USA, 2015) and R: A language and environment for statistical computing v3.6.3 (64b) (R Foundation for Statistical Computing, Vienna, Austria, 2016) were used [12].

## 3. Results

### 3.1. Participants and Lesions

Between July 2014 and June 2016, a total of 2123 lesions >10 mm that were not obvious cancer (type 0 in Paris classification) were collected from 1634 consecutive patients. After excluding pedunculated lesions and those measuring less than 20 mm, 542 superficial lesions from 517 patients were included in the analysis. The blinded and centralised histological assessment was performed from July 2016 to March 2017. Patients’ mean age was 68.3 (SD 10.5) years, and 313 (60.5%) were male. The colonoscopy indication was asymptomatic screening in 321 (62.1%) patients and clinical symptoms in 196 (37.9% patients). Lesion characteristics are shown in Table 1.

### 3.2. Lesion Characteristics Associated with Intramucosal Neoplasia

Bivariate analysis showed that size, right-sided location, LSL-granular (G) homogeneous type, LSL-non granular (NG) flat elevated type, the absence of most gross morphologic malignant features (non-lifting sign, chicken skin sign, depressed areas, fold convergence, induration, ulceration), and NICE 1 and 2 lesions were associated with intramucosal neoplasia (Table 1).

### 3.3. Conditional Inference Tree for Identifying Intramucosal Neoplasia

**Figure 1:**
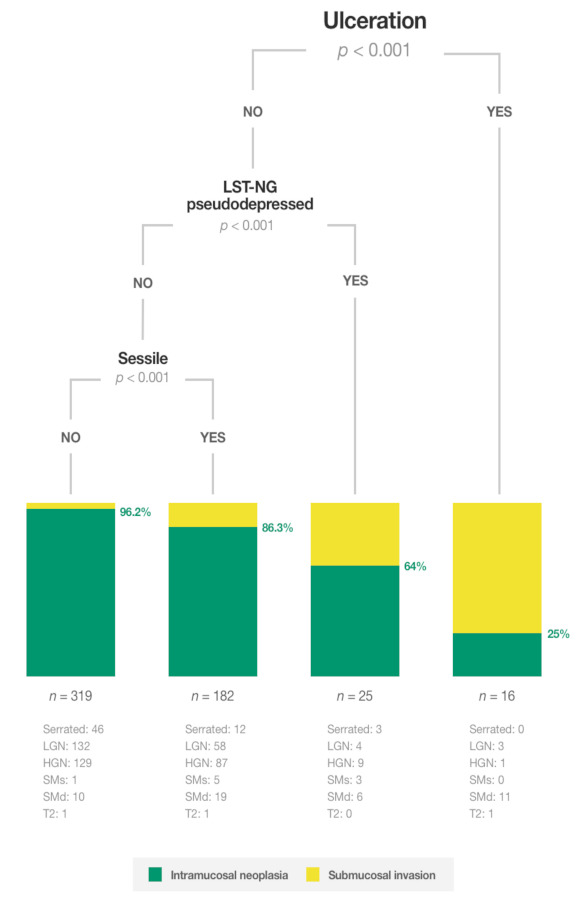
Conditional inference tree for identifying intramucosal neoplasia.

Intramucosal neoplasia was present in 484 of 542 (89.3%) lesions. Performing a CTREE algorithm with the full sample (all the registered variables) produced a very stable tree (Figure 1). Ulceration, pseudodepressed type and sessile morphology changed the accuracy for predicting intramucosal neoplasia. In ulcerated lesions, the probability of intramucosal neoplasia was 25% (95%CI: 8.3–52.6%; *p* < 0.001). In non-ulcerated lesions, the probability of intramucosal neoplasia rose in lateral spreading lesions (LST) non-granular (NG) pseudodepressed type lesions to 64.0% (95%CI: 42.6–81.3%; *p* < 0.001). Sessile morphology also raised the probability of intramucosal neoplasia to 86.3% (95%CI: 80.2–90.7%; *p* < 0.001). In the remaining 319 (58.9%) non-ulcerated lesions that showed LST-Granular (G) homogeneous type, LST-G nodular-mixed type, and LST-NG flat elevated morphology, the probability of intramucosal neoplasia was 96.2% (95%CI: 93.5–97.8%; *p* < 0.001).

### 3.4. Conditional Inference Tree for Identifying Shallow sm Invasion

No stable CTREE algorithm was able to identify nine out of 542 lesions with shallow sm invasion.

### 3.5. Conditional Inference Tree for Identifying Deep sm Invasion

**Figure 2:**
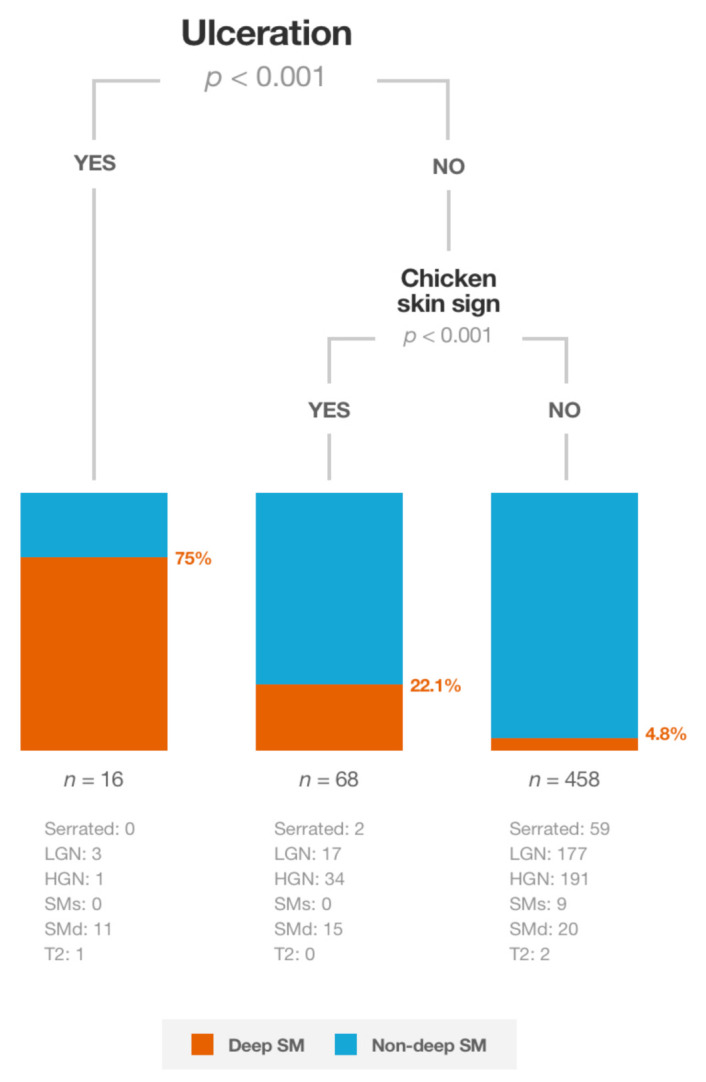
Conditional inference tree for predicting deep submucosal invasion.

Performing a CTREE algorithm with the full sample showed that ulceration was the variable that most accurately identified lesions with deep sm invasion (Figure 2). In ulcerated lesions, the probability of deep sm invasion was 75.0% (95%CI: 50.5–89.8%; *p* < 0.001). In the absence of ulceration, deep sm invasion was 22.1% (95%CI: 13.8–33.3%; *p* < 0.001) in lesions with the chicken skin sign, and 4.8% (95%CI: 3.2–7.2%; *p* < 0.001) if neither of these features was present.

## 4. Discussion

This is the first study to develop a classification system with a conditional inference tree based on endoscopic features to identify intramucosal neoplasia in non-pedunculated lesions ≥20 mm, assessed prospectively and in situ by western endoscopists with NBI and without magnification. Non-ulcerated LST-G type and LST-NG flat elevated lesions represented 58.8% of all non-pedunculated lesions ≥20 mm and were associated with a high probability of intramucosal neoplasia (96.2%). Therefore, these lesions are a priori suited to treatment with piecemeal EMR. However, for all the remaining lesions, further diagnostic techniques like observation with magnification, and advanced diagnostic +/− therapeutic procedures like ESD or surgery should be considered, depending on the resources available and patients’ morbidity and preferences.

These results are consistent with those of previous studies where size, location, different morphologies and gross morphological malignant features were associated with sm invasion [9,10,11]. The study conducted by Backes et al. [9] used a Lasso model to analyse the features of 347 lesions and identified the probability of sm invasion in 128 categories. In that study, there were few lesions with a low risk of sm invasion (the number was not mentioned), and the 95% confidence intervals were wide due to the low number of lesions in each category. In the study by Burgess et al. [11], multiple logistic regression with backward stepwise variable selection was used to identify the independent predictors of sm invasion. As a result, few lesions are classified as unlikely to present sm invasion. In our study, the combination of all these characteristics analysed by a conditional inference tree selected only three variables and covered a large proportion of lesions (58.8%) by a simple algorithm. In the organisation of a multistep system for the homogenisation of the diagnosis and treatment of colorectal lesions, this might be the first step for selecting lesions suitable for treatment by piecemeal EMR by non-reference endoscopists and centres. In the remaining lesions, local committees that consider patients’ morbidity and preferences, and the resources available at reference centres, should decide whether the lesions require further diagnostic techniques like observation with magnification, and advanced diagnostic +/− therapeutic procedures like ESD or surgery.

Surprisingly, in our study, there were four (25%) ulcerated lesions without sm invasion (three with LGN and one with HGN). Two of these lesions were located in the rectum. By definition, ulceration is an amorphous surface (Kudo pit pattern Vn) and an avascular area (JNET 3 or Sano IIIB), clearly associated with deep sm invasion [19,20]. Although no photodocumentation was required in our study, we suggest two possible reasons for this inconsistency: (1) sometimes the mucous can mimic ulceration and its removal is challenging; (2) some lesions located in the rectum close to the anus may be ulcerated if a prolapse syndrome exists. Therefore, we believe that these two points should be considered when ulceration is presumed, but this type of lesion can be biopsied and referred to surgery (Figure 2).

In the absence of ulceration, the probability of intramucosal neoplasia was 64.0% in LST-NG pseudodepressed lesions. Subsequently, piecemeal EMR should be avoided in these lesions if magnification or ESD is available at the same or a reference centre.

In sessile lesions, the probability of intramucosal neoplasia was 86.3%. This is consistent with previous studies which found covert sm invasion in 10.5% of the lesions. However, the diagnostic accuracy of optical magnification also tends to fall in large protruded lesions. In a retrospective study by Sakamoto et al. [21], 28% of 112 large protruded lesions that were initially treated by ESD (with no invasive pattern) showed deep sm invasion, including seven (6%) T2 lesions and one (1%) T3. Therefore, different diagnostic and treatment options should be discussed carefully in large protruded lesions.

For the remaining non-ulcerated lesions (LST-G homogeneous and nodular-mixed type and LST-NG flat elevated), piecemeal EMR seems to be a reasonable option if no magnification is available at the centre, since the probability of inconclusive histology due to sm invasion is very low (3.8%).

Not surprisingly, the CTREE did not find the NICE classification to be useful for predicting intramucosal neoplasia (LGN or HGN). NICE 2 without magnification was designed to predict LGN, HGN and shallow submucosal invasion. Therefore, the pointlessness of the NICE classification for ruling out shallow submucosal invasion and choosing piecemeal EMR is consistent with the previous literature [22]. By contrast, the JNET classification suggests that JNET 2A lesions should be treated with piecemeal EMR because LGN is predicted, and JNET 2B lesions should be treated with en bloc resection because HGN or shallow sm invasion is the most likely histology [7]. Given that there is no need to evaluate R0 margins or risk factors for LNM in lesions with HGN, there are two main reasons for including these lesions in the group that should be treated en bloc when evaluated with zoom. The first is that several studies conducted with magnification have been unable to optically distinguish HGN from shallow sm invasion [23]. The second is that advanced cancer recurrence has been observed in two Tis out of 153 Tis/SMs lesions removed by piecemeal EMR. The authors suggest an inaccurate initial histological diagnosis of HGN [24]. Another recent study also showed that 6 out of 138 (4.3%) lesions with high-grade dysplasia removed in a piecemeal fashion led to a local recurrence as malignancy [25]. In our study, we explored the usefulness of lesion characteristics without magnification for predicting LGN (Supplementary Table S1 and Supplementary Figure S1). Although size, location, morphology, absence of some gross morphological malignant features and the NICE classification were associated with LGN, the conditional inference tree found intermediate risks for detecting LGN, thus rendering the process unreliable for making decisions. Therefore, considering the minimal clinical implications and the limited diagnostic accuracy without magnification, predicting LGN should not be the aim when lesions are assessed without zoom.

**Figure 3:**
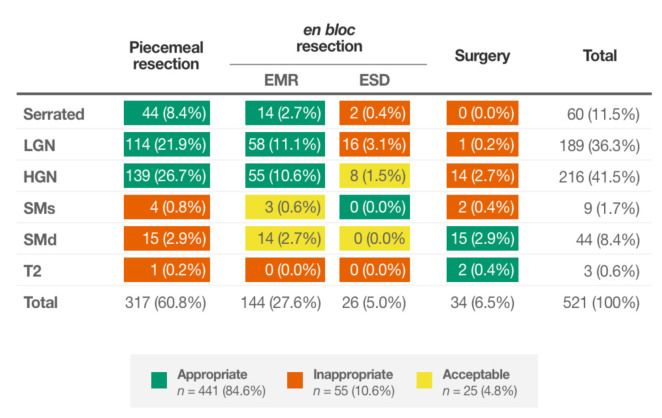
Lesion histology according to the treatment performed in clinical practice.

The clinical consequences of these findings were also explored. Piecemeal EMR was performed in 317 (60.8%) of the lesions, and sm invasion was found in 20 (6.3%) of them (Figure 3). The algorithm suggested the performance of piecemeal EMR similarly in 319 (58.9%) lesions, but inconclusive histology due to sm invasion would have been found in 12 (3.8%). Moreover, among 521 lesions where the local resection technique (en bloc or piecemeal) was recorded, if piecemeal EMR had been conducted in the 315 lesions suggested by the algorithm: (1) 20 (6.3%) ESD would have been avoided (one serrated histology, 14 with LGN and five with HGN) (2) 10 (3.2%) surgeries would not have been initially performed (eight with HGN and two with deep invasion), while the number of lesions with inconclusive histology due to sm invasion would have been limited to 12 (3.8%). This highlights the room for improvement in our routine clinical practice and the potential usefulness of the algorithm.

Our study is not without limitations. First, this is a post-hoc analysis of prospectively collected data designed for predicting deep sm invasion in colorectal polyps. Therefore, the association between these characteristics and the absence of sm invasion should be considered carefully as a real feature for predicting intramucosal neoplasia. Second, although the CTREE algorithm identified significant variables, the study may not possess enough statistical power to detect other significant variables. This point is especially relevant in the rectum because (1) this and other studies have found a much higher risk of sm invasion in this site; (2) inconclusive histology due to a piecemeal EMR may lead to surgical rescue treatments with non-negligible comorbidity rates in lesions with sm invasion; (3) en bloc diagnostic techniques like ESD and transanal minimally invasive surgery are widely available and may provide a more precise histology diagnosis. Third, the algorithm should be subsequently validated and the improvement of clinical significant outcomes should be assessed.

## 5. Conclusions

In conclusion, non-ulcerated LST-G type and LST-NG flat elevated lesions represent 58.8% of all non-pedunculated lesions ≥20 mm and are associated with a low risk of sm invasion (3.8%). Therefore, these lesions can be directly treated by piecemeal EMR. However, for the rest of the non-pedunculated polyps ≥20 mm (41%) further diagnostic techniques like observation with magnification are recommended for better selection of those that would benefit from an en bloc resection. In this scenario, ESD might also be considered as a diagnostic tool to provide high-quality specimens for further histological assessment.

## Figures and Tables

**Table 1:** Lesion characteristics according to the presence of intramucosal neoplasia (m) or submucosal invasion (sm).

Lesion Characteristics	Overall *n* = 542	m *n* = 484	sm *n* = 58	*p* Value
**Size**, mm, median [25th percentile–75th percentile]	28.0 [20.0–35.0]	26.5 [20.0–35.0]	30.0 [25.0–40.0]	0.023
**Size**, mm				0.138
20–24	162 (29.9%)	152 (31.4%)	10 (17.2%)	
25–29	111 (20.5%)	99 (20.5%)	12 (20.7%)	
30–34	111 (20.5%)	97 (20.0%)	14 (24.1%)	
35–39	51 (9.4%)	46 (9.5%)	5 (8.6%)	
≥40	107 (19.7%)	90 (18.6%)	17 (29.3%)	
**Location**				<0.001
Right colon	314 (57.9%)	296 (61.2%)	18 (31.0%)	
Left colon	100 (18.5%)	81 (16.7%)	19 (32.8%)	
Rectum	128 (23.6%)	107 (22.1%)	21 (36.2%)	
**Morphology**				
**Polypoid**				
Sessile (0-Is)	192 (35.4%)	159 (32.9%)	33 (56.9%)	<0.001
**Non-polypoid**				
Homogeneous type (LST-G IIa)	76 (14.0%)	75 (15.5%)	1 (1.7%)	0.004
Nodular mixed type (LST-G IIa+Is)	96 (17.7%)	87 (18.0%)	9 (15.5%)	0.643
Elevated type (LST-NG IIa)	150 (27.7%)	146 (30.2%)	4 (6.9%)	<0.001
Pseudodepressed type (LST-NG IIa+IIc)	28 (5.2%)	17 (3.5%)	11 (19.0%)	<0.001
**Gross morphological malignant features**				
Non-lifting sign *	32 (6.5%)	24 (5.3%)	8 (19.5%)	<0.001
Chicken skin sign	73 (13.5%)	54 (11.2%)	19 (32.8%)	<0.001
Edge retraction	14 (2.6%)	12 (2.5%)	2 (3.4%)	0.654
Depressed areas	74 (13.7%)	47 (9.7%)	27 (46.6%)	<0.001
Folds convergence	17 (3.1%)	12 (2.5%)	5 (8.6%)	0.027
Induration	16 (3.0%)	7 (1.4%)	9 (15.5%)	<0.001
Ulceration	16 (3.0%)	4 (0.8%)	12 (20.7%)	<0.001
Polyp over polyp	19 (3.5%)	16 (3.3%)	3 (5.2%)	0.709
**NICE**				<0.001
NICE 1	44 (8.1%)	43 (8.9%)	1 (1.7%)	
NICE 2	445 (82.1%)	422 (87.2%)	23 (39.7%)	
NICE 3	53 (9.8%)	19 (3.9%)	34 (58.6%)	

* Among 496 lesions (elevation was not attempted in 46).

## Data Availability

The data presented in this study are available on request from the corresponding author. The data are not publicly available due to the spanish data protection law that restricts access to pseudo-anonymized personal data.

## Supplementary Materials

The following are available online at https://www.mdpi.com/article/10.3390/cancers13215302/s1, Figure S1: Algorithm for predicting LGN, Table S1: Lesions characteristics according to the presence of LGN or HGN/SM; Document S1: AEG Red Cap; Document S2.: Conditional inference tree methodology.
