# Supplementary material for: Compound Endoscopic Morphological Features for Identifying Non-Pedunculated Lesions ≥20 mm with Intramucosal Neoplasia
Source: Cancers (Basel). 2021 Oct 22;13(21):5302. doi: 10.3390/cancers13215302 (PMC8582371; doi:10.3390/cancers13215302)
Supplement: Supplementary file 1 [file cancers-13-05302-s001.zip › Supplementary document S1.pdf]

Supplementary document S1

AEG is a non-profit Scientific and Medical Society focused on Gastroenterology, and it provided this service free of charge, with the sole aim of promoting independent investigator-driven research. REDCap (Research Electronic Data Capture) is a secure, web-based application designed to support data capture for research studies, providing 1) an intuitive interface for validated data entry; 2) audit trails for tracking data manipulation and export procedures; 3) automated export procedures for seamless data downloads to common statistical packages; and 4) procedures for importing data from external sources.
