# Supplementary material for: Compound Endoscopic Morphological Features for Identifying Non-Pedunculated Lesions ≥20 mm with Intramucosal Neoplasia
Source: Cancers (Basel). 2021 Oct 22;13(21):5302. doi: 10.3390/cancers13215302 (PMC8582371; doi:10.3390/cancers13215302)
Supplement: Supplementary file 1 [file cancers-13-05302-s001.zip › Supplementary document S2 .pdf]

**Supplementary document S2. Inference tree methodology**

CTREE is an unbiased recursive binary partitioning algorithm that solves both the overfitting and selection bias. "Statistical models that regress the distribution of a response variable on the status of multiple covariates are tools for handling two major problems in applied research: prediction and explanation. The function space represented by regression models focusing on the prediction problem may be arbitrarily complex; indeed, 'black box' systems like support vector machines or ensemble methods are excellent predictors. In contrast, regression models appropriate for gaining insight into the mechanism of the data generating process are required to offer a human readable representation. Generalized linear models or the Cox model are representatives of regression models where parameter estimates of the coefficients and their distribution are used to judge the relevance of single covariates".[23]

Recursive binary partitioning (RBP) algorithms work in a significant different way than most traditional regression ones. While parametric algorithms like ordinary least squares (OLS) or logistic regression are designed to estimate influence parameters across independent variables in the form of partial correlation coefficients or logarithms of odd's ratios, tree-like algorithms based on RBP search to split the sample at all variables and points possible to maximize predictive power.

A critical difference between methods lays in the variable inclusion process. Most parametric regression methods are not designed for variable selection or feature engineering. Variables or covariates must be prespecified and explicitly plugged in a matrix form to be able to estimate the desired parameters. In contrast, RBP methods, by nature, select automatically the most relevant features in any functional form to maximize predictive power.

A generic representation of an RBP follows (as presented in Hothorn et al. 2006):

1. For case weights **w** test the global null hypothesis of independence between any of the *m* covariates and the response. Stop if this hypothesis cannot be rejected. Otherwise select the *j**th covariate *Xj** with strongest association to Y.

2. Choose a set $A^* \subset Xj^*$ to split $Xj^*$ into two disjoint sets $A^*$ and $Xj^* \setminus A^*$. The case weights $\mathbf{w}_{\text{left}}$ and $\mathbf{w}_{\text{right}}$ determine the two subgroups with $\mathbf{w}_{\text{left},i} = \mathbf{w}_i \mathrm{I}(X_{j*i} \in A^*)$ and $\mathbf{w}_{\text{right},i} = \mathbf{w}_i \mathrm{I}(X_{j*i} \in A^*)$ for all $i = 1, \ldots, n$ ($\mathrm{I}(\cdot)$ denotes the indicator function).

3. Recursively repeat steps 1 and 2 with modified case weights $\mathbf{w}_{\text{left}}$ and $\mathbf{w}_{\text{right}}$, respectively.
