# Supplementary material for: Compound Endoscopic Morphological Features for Identifying Non-Pedunculated Lesions ≥20 mm with Intramucosal Neoplasia
Source: Cancers (Basel). 2021 Oct 22;13(21):5302. doi: 10.3390/cancers13215302 (PMC8582371; doi:10.3390/cancers13215302)
Supplement: Supplementary file 1 [file cancers-13-05302-s001.zip › Supplementary Table S1.pdf]

Supplementary Table S1. Lesions characteristics according to the presence of LGN or HGN/SM

**Lesions characteristics**	**Overall** n=542	**LGN** n=258	**HGN/SM** n=284	*P* value
**Size**, mm, median [25th percentile – 75th percentile]	28.0 [20.0-35.0]	25.0 [20.0-30.0]	30.0 [25.0-40.0]	<0.001
**Size**, mm				<0.001
20-24	162 (29.9%)	95 (36.8%)	67 (23.6%)	
25-29	111 (20.5%)	55 (21.3%)	56 (19.7%)	
30-34	111 (20.5%)	56 (21.7%)	55 (19.4%)	
35-39	51 (9.4%)	24 (9.3%)	27 (9.5%)	
≥40	107 (19.7%)	28 (10.9%)	79 (27.8%)	
**Location**				<0.001
Right colon	314 (57.9%)	177 (68.6%)	137 (48.2%)	
Left colon	100 (18.5%)	39 (15.1%)	61 (21.5%)	
Rectum	128 (23.6%)	42 (16.3%)	86 (30.3%)	
**Morphology**				
**Polypoid**				
Sessile (0-Is)	192 (35.4%)	71 (27.5%)	121 (42.6%)	<0.001
**Non-polypoid**				
Homogeneous type (LST-G IIa)	76 (14.0%)	38 (14.7%)	38 (13.4%)	0.652
Nodular mixed type (LST-G IIa+Is)	96 (17.7%)	39 (15.11%)	57 (20.1%)	0.131
Elevated type (LST-NG IIa)	150 (27.7%)	102 (39.5%)	48 (16.9%)	<0.001
Pseudodepressed type (LST-NG IIa+IIc)	28 (5.2%)	8 (3.1%)	20 (7.0%)	0.038
**Gross morphological malignant features**				
Non-lifting sign*	32 (6.5%)	13 (5.3%)	19 (7.6%)	0.294
Chicken skin sign	73 (13.5%)	19 (7.4%)	54 (19%)	<0.001
Edge retraction	14 (2.6%)	4 (1.6%)	10 (3.5%)	0.149
Depressed areas	74 (13.7%)	20 (7.8%)	57 (19%)	<0.001
Folds convergence	17 (3.1%)	5 (1.9%)	12 (4.2%)	0.127
Induration	16 (3.0%)	2 (0.8%)	14 (4.9%)	0.004
Ulceration	16 (3.0%)	3 (1.2%)	13 (4.6%)	0.019
Polyp over polyp	19 (3.5%)	3 (1.2%)	16 (5.6%)	0.005
**NICE**				<0.001
NICE 1	44 (8.1%)	41 (15.9%)	3 (1.1%)	
NICE 2	445 (82.1%)	213 (82.6%)	232 (81.7%)	
NICE 3	53 (9.8%)	4 (1.6%)	49 (17.3%)	

* Among 496 lesions (elevation was no attempted in 46 lesions).
