# Supplementary figures and images for: Compound Endoscopic Morphological Features for Identifying Non-Pedunculated Lesions ≥20 mm with Intramucosal Neoplasia
Source: Cancers (Basel). 2021 Oct 22;13(21):5302. doi: 10.3390/cancers13215302 (PMC8582371; doi:10.3390/cancers13215302)

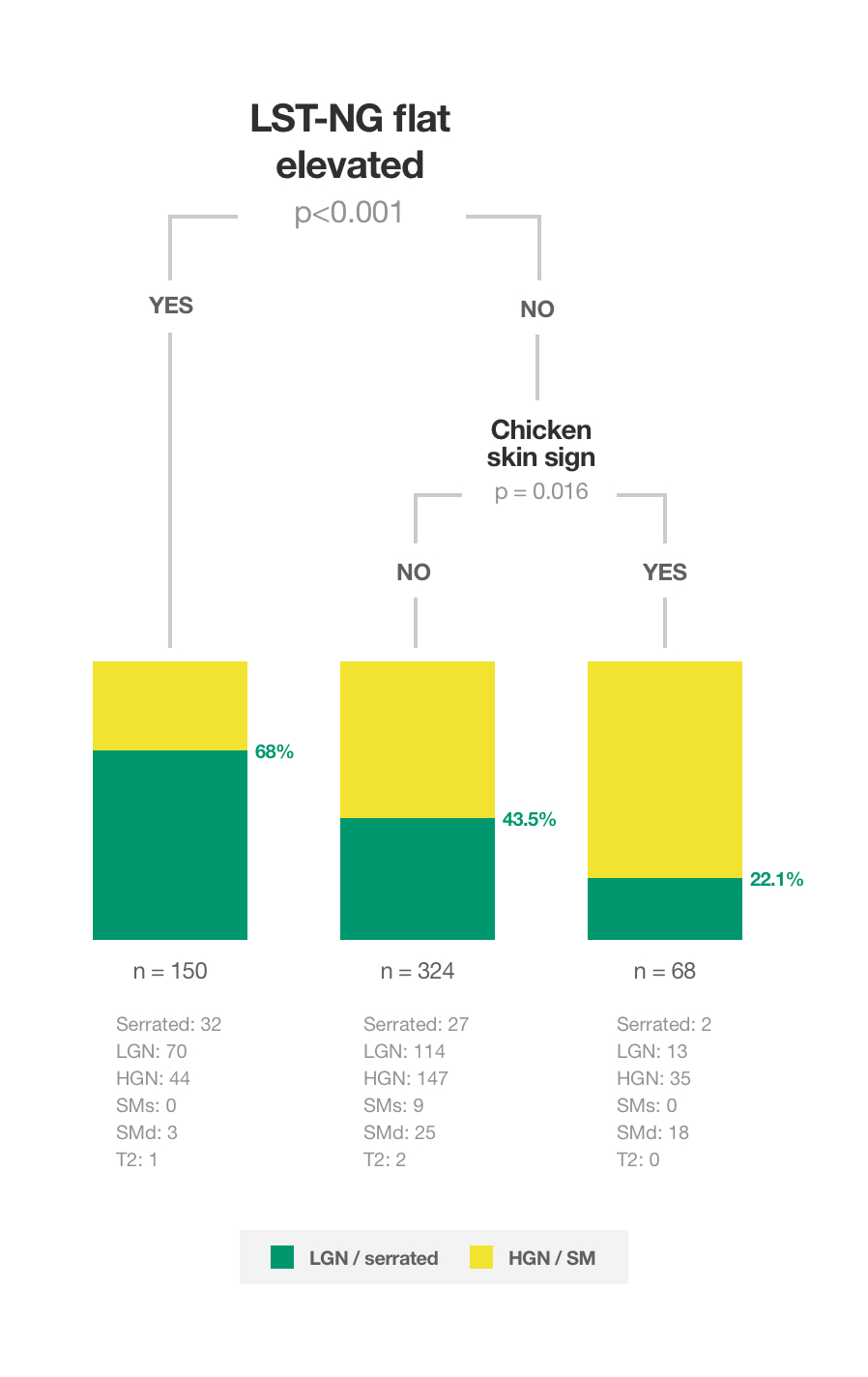

Supplement: Supplementary file 1 [file cancers-13-05302-s001.zip › Supplementary Figure S1.jpg]
